# Is location a significant parameter in the layer dependent dissection properties of the aorta?

**DOI:** 10.1007/s10237-022-01627-9

**Published:** 2022-09-03

**Authors:** Itziar Ríos-Ruiz, Miguel Ángel Martínez, Estefanía Peña

**Affiliations:** 1 Aragón Institute of Engineering Research (I3A), University of Zaragoza, C/ Mariano Esquillor, s/n, Zaragoza, 50018 Spain; 2 Centro de Investigación Biomédica en Red en Bioingeniería, Biomateriales y Nanomedicina (CIBER-BBN), Zaragoza, Spain

**Keywords:** Aortic dissection, Delamination tests, T-peel test, Mixed-mode peel test, Porcine aorta, Tissue characterisation

## Abstract

Proper characterisation of biological tissue is key to understanding the effect of the biomechanical environment in the physiology and pathology of the cardiovascular system. Aortic dissection in particular is a prevalent and sometimes fatal disease that still lacks a complete comprehension of its progression. Its development and outcome, however, depend on the location in the vessel. Dissection properties of arteries are frequently studied via delamination tests, such as the T-peel test and the mixed-mode peel test. So far, a study that performs both tests throughout different locations of the aorta, as well as dissecting several interfaces, is missing. This makes it difficult to extract conclusions in terms of vessel heterogeneity, as a standardised experimental procedure cannot be assured for different studies in literature. Therefore, both dissection tests have been here performed on healthy porcine aortas, dissecting three interfaces of the vessels, i.e., the intima-media, the media-adventitia and the media within itself, considering different locations of the aorta, the ascending thoracic aorta (ATA), the descending thoracic aorta and the infrarenal abdominal aorta (IAA). Significant differences were found for both, layers and location. In particular, dissection forces in the ATA were the highest and the separation of the intima-media interface required significantly the lowest force. Moreover, dissection in the longitudinal direction of the vessel generally required more force than in the circumferential one. These results emphasise the need to characterise aortic tissue considering the specific location and dissected layer of the vessel.

## Introduction

Arterial dissection is a vascular pathology that occurs in the main vessels of the cardiovascular system, namely, the aorta, the carotid arteries and the coronary arteries (Tong et al. 2016). This pathology is characterised by the propagation of a tear throughout the walls of a vessel (Nienaber et al. 2016; Tong et al. 2016). Due to this propagation, a false lumen can be created, which could imply a narrowing or even a collapse of the actual lumen (Criado 2011), or the formation of blood clots, that could occlude more distal vessels. The dissection can also propagate further and reach the adventitial layer, where it can cause the rupture of the vessel with an often fatal outcome (Kouchoukos and Dougenis 1997; Nienaber et al. 2016; Schievink 2001; Tong et al. 2016). Acute aortic dissection involving the ascending part of the aorta has an in-hospital mortality of up to 50% in the first hours (Fleischmann et al. 2022; Howard et al. 2013; Silaschi et al. 2017).

Although the specific initial cause is still unknown, two mechanisms have been suggested as triggering factors and are widely accepted: i) a tear in the intimal layer of the vessel wall, which can happen spontaneously, in an already damaged intima, or provoked by external trauma, e.g., in medical interventions and ii) the rupture of the vasa vasorum, causing a weakening of the vessel wall and an intramural haematoma that can progress through the wall (Nienaber et al. 2016). Depending on the location and comorbidities, one of these two explanations has been found to be prevalent. For example, a study of 505 cases of dissected aneurysms in the aorta determined that there were signs of an initial intimal tear in 96% of cases (Hirst et al. 1958), whereas spontaneous coronary artery dissection is characterised by the presence of an intramural haematoma (Lewey et al. 2022). The risk factors associated with the weakening or damaging of the intima so far are age, hypertension, smoking and congenital and genetic disorders, among others (Evangelista et al. 2018; Howard et al. 2014; Nienaber et al. 2016; Sherifova and Holzapfel 2019). Nevertheless, current investigation of arterial dissection has focused on its propagation instead of its initiation, as patients arise once the pathology has started (Rajagopal et al. 2007).

Dissection in the aorta can happen throughout the entire vessel and, depending on the area in which it occurs, the outcome is frequently different (Howard et al. 2013; Silaschi et al. 2017). In fact, the current classification of aortic dissections, the Stanford Classification (Daily et al. 1970), sorts the dissections only considering the location of the aorta in which they appear. Stanford type A dissections involve the ascending aorta, whereas Stanford type B dissections do not (Criado 2011). Dissections that appear in the ascending part of the aorta, which account for around 60-70% of the cases (Evangelista et al. 2018; Fleischmann et al. 2022; Hagan et al. 2000; Landenhed et al. 2015), tend to need surgical intervention due to their severity, as they can involve failure in other surrounding vessels (like the coronary arteries). On the other hand, dissections occurring in the descending thoracic or the abdominal aorta are more commonly medically treated as they are most likely to turn chronic, but they can also need endovascular intervention (Evangelista et al. 2018; Nienaber et al. 2016). It is, therefore, a pathology that depends on location.

In order to improve the understanding of the dissection and fracture behaviour of the vessels, several methodologies have been developed. Tensile tests and the determination of the ultimate tensile strength of the vessel wall in the three main directions—longitudinal, circumferential and radial—had been used as a way of analysing failure properties of the vessels (Manopoulos et al. 2018; Mohan and Melvin 1982; Peña et al. 2019; Purslow 1983; Sommer et al. 2008; Xuan et al. 2021). Subsequently, centering the focus on arterial dissection, delamination tests were introduced for vascular tissue as a way of reproducing the propagation of a tear in vitro. In particular, a T-peel test, which involves a mode I of fracture, was proposed by Sommer et al. (2008). This test consists in the progressive separation of the layers of a specimen by normally pulling from the flaps of two layers. In addition, a mixed-mode peel test was developed as a more physiological way of dissecting two layers, as it involves a mixed mode of fracture, more similar to the real scenario (Leng et al. 2018). In this case, the layers are dissected by pulling from one flap in parallel to the plane of dissection. Both peel tests are described in detail in Sect. 2.2. Several studies have based their dissection investigations in these peeling tests. Whereas most work has focused on aortic dissection (Angouras et al. 2019; Horný et al. 2022; Kozuń 2016; Kozuń et al. 2018; Leng et al. 2018; Myneni et al. 2020; Noble et al. 2016; Pasta et al. 2012; Sokolis and Papadodima 2022; Sommer et al. 2008; Tong et al. 2014; Wang et al. 2021), dissection in the carotid and coronary arteries has also been studied (Tong et al. 2011; Wang et al. 2013). Regarding diseased conditions, several studies have focused on the dissection properties of aneurysmal tissue (Angouras et al. 2019; Pasta et al. 2012; Sommer et al. 2016), since a ruptured aneurysm is not rare and is mostly fatal (Assar and Zarins 2009). So far, aortic dissection studies have commonly focused on one specific location of the aorta (Leng et al. 2018; Noble et al. 2016; Purslow 1983; Sommer et al. 2008; Wang et al. 2021) and have dissected either the medial layer within itself (Horný et al. 2022; Myneni et al. 2020; Sokolis and Papadodima 2022) or the separation of the two interfaces (intima-media and media-adventitia) (Kozuń 2016; Kozuń et al. 2018), sometimes comparing between healthy and diseased conditions (Angouras et al. 2019; Kozuń 2016; Kozuń et al. 2018; Pasta et al. 2012). Recently, Horný et al. (2022) and Sokolis and Papadodima (2022) evaluated the dissection behaviour of the human aorta considering the different locations throughout the vessel. However, the number of studies is still low and the conditions in each work sometimes vary, therefore the variation in the results is rather large (Sherifova and Holzapfel 2019). To our knowledge, no study has reported yet the dissection behaviour through different locations of the aorta in both directions and between all interfaces, nor performing both dissection tests. Considering the contrasting progression and outcome of this disease in the different locations of the aorta, it is of great interest to perform a dissection study on the entire vessel.

Therefore, a full dissection study in a porcine aorta is here presented. The study includes the three main zones of the aorta, i.e., the ascending thoracic aorta (ATA), the descending thoracic aorta (DTA) and the infrarenal abdominal aorta (IAA). In all these areas, peel tests were performed to separate three interfaces of the vessel, the intima-media, the media-adventitia and within the media, in the longitudinal and circumferential directions of the vessel. Two different tests, the T and mixed-mode peel tests, have been performed for each condition, and the results are presented in terms of mean peeling force per width, dissection energy per reference area and separation distance at damage initiation.

## Materials and methods

### Obtention of samples

In the present study, a total of 9 healthy porcine aortas were harvested postmortem from female pigs. The swines were 3.5 ± 0.45 months old and weighed 45 ± 5 kg. They had been sacrificed for different studies that do not interfere with the aorta or the circulatory system, therefore no animal was killed specifically for these experiments. The experiments were approved by the Ethical Committee for Animal Research of the University of Zaragoza, with code PI36/20, and all procedures were carried out in accordance with the “Principles of Laboratory Animal Care” (86/609/EEC Norm). The animals were sacrificed under general anaesthesia through an intravenous injection of potassium chloride and sodium thiopental, and the aortas were harvested by skilled veterinarians. All vessels collected were complete, including the three zones of study: ATA, DTA and IAA, except for 2 arteries, in which the portion of the IAA was missing. Once harvested, the whole arteries were kept frozen at −80 °C to assure a proper preservation, and thawed 24 hours before the tests at 4 °C. The specimens were cut in rectangular shapes of a dimension of 20 x 5 mm, approximately. The width, height and thickness of the specimens was subsequently measured. Table 1 shows the mean thickness according to location. Until testing, the rectangular samples were kept in ion-free physiological saline solution (PSS, 0.9 % NaCl) at 4 °C. All experiments were performed within 48 hours after the defrosting of the samples.

**Table 1:** Thickness of the specimens as function of location in the aorta

Location	Thickness (mm)
ATA	2.17 ± 0.54 (*n* = 108)
DTA	1.99 ± 0.46 (*n* = 105)
IAA	1.12 ± 0.23 (*n* = 78)

### Experimental procedure

In each location of the artery, a total of 12 specimens were obtained, 6 strips per direction (longitudinal and circumferential). These 6 specimens were divided into two sets of 3 samples, one set for the T-peel test and the other for the mixed-mode peel test. In these sets, there is one specimen for each separation layer, i.e. intima-media (IM), media-adventitia (MA) and within the media (M). An initial incision of around 5 mm in length was performed in order to assure that the separation occurs between the layers of interest. In case the initial incision was faulty or the dissection test was not successful, some extra specimens were cut to help complete the set where possible.

**Fig. 1:**
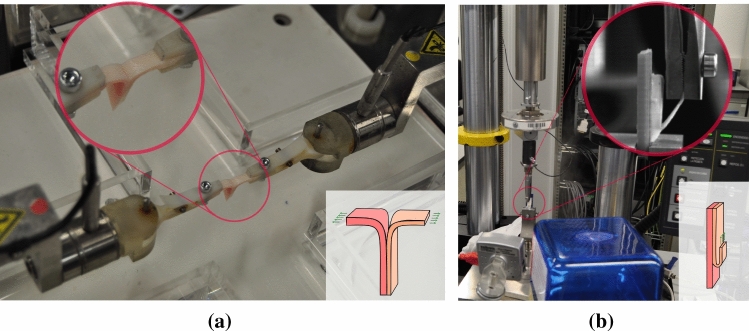
Experimental setup and descriptive outline of the experimental tests. (**a**) shows the dissection of a sample by means of the T-peel test in the Instron BioPuls™ low-force planar-biaxial Testing System. (**b**) shows the dissection via the mixed-mode peel test in the high precision drive Instron Microtester 5548 system

In the T-peel test, the two tongues of the specimen are gripped by two moving clamps. These clamps move in opposite directions at a speed of 1 mm/min each, which entails a total testing speed of 2 mm/min, separating the layers of the specimen in the direction normal to the interface plane. The test ends after 20 mm of separation or the complete dissection of the sample. An outline of the T-peel test is shown in Fig. 1a. In the mixed-mode peel test, one side of the sample is glued to a clamp plate and completely fixed. This is always the intimal side as its surface is softer and allows for a better attachment. Therefore, it is only one flap that is gripped by a moving clamp, which moves at a speed of 1 mm/min in the parallel direction of the specimen, see Fig. 1b. The T-peel and mixed-mode peel tests were carried out in the Instron BioPuls™ low-force planar-biaxial Testing System and the high precision drive Instron Microtester 5548 system adapted for biological specimens, respectively. Load cells of 10 N were used (Instron 2530-428), with an accuracy of 2.5 mN and a displacement resolution of 0.015 mm. The experiments were performed at room temperature and samples were either submerged or humidified with PSS in the T-peel and the mixed-mode peel test, respectively, to assure proper hydration throughout the experiments.

### Histology

In order to microstructurally check the proper separation of the layers, histologies were performed in the dissected tissue. Prepared samples were processed in the histological laboratory. They were washed with normal saline solution at room temperature, subsequently fixed in 10% buffered formalin and embedded in paraffin following standard procedures. The histology blocks were sectioned at 5 $\mu$m and stained with Masson’s trichrome stain as it allows to identify the general structure of the different layers of the vessel (muscle fibres stained in red, cell nuclei in dark purple and connective tissue—collagen and elastin—in light green). Samples came from T-peel tests that were not performed until complete separation, in order to be able to check both interfaces in the histologies.

### Mean peeling force/width, dissection energy and separation distance at damage initiation

The force vs displacement curves can be extracted from the experimental peeling tests. The data are later processed and the values of force are divided by the width of the specimen to avoid the effect of this dimension in the results. Mean force/width and standard deviation of the tests is calculated throughout the separation of the specimens.

**Fig. 2:**
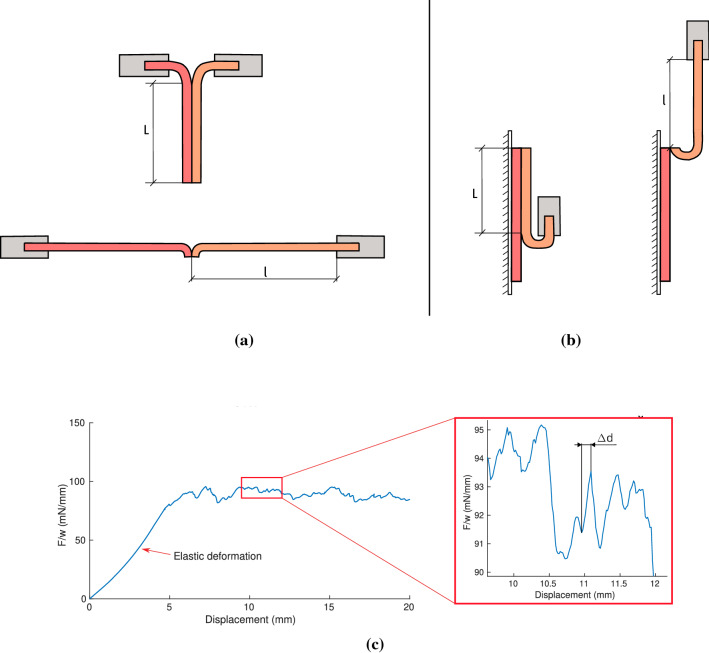
Outline of the considered dimensions to calculate the dissection energy in the T-peel test (**a**) and mixed-mode peel test (**b**). (**c**) shows an example of the obtention of displacement increments $\Delta d$ in the force/width curves

The dissection energy or critical energy release rate ($G_c$) for both peel tests is calculated following the proposed method by Sommer et al. (2008). Briefly, the dissection energy per reference area is the difference between the external work, $W_{ext}$, and the internal elastic energy, $W_{elas}$, i.e., $G_c=(W_{ext}-W_{elas})/L$. *L* is the initial length of the interface to be dissected, as shown in Fig. 2a and b. The external work is defined by $W_{ext}=2Fl$ in the T-peel test, and by $W_{ext}=F(L+l)$ in the mixed-mode peel test, where *F* is the force applied to dissect the specimen per reference width and *l* is the length of the dissected specimen right before complete separation, see Fig. 2a and b. Assuming a linear relationship between the first Piola-Kirchhoff stress and the related stretch (Myneni et al. 2020; Sommer et al. 2008), the elastic energy can be defined as $W_{elas}=F(l-L)$.

The separation distance at damage initiation ($\delta _0$) is obtained from the experimental curves following the approximation proposed by Wang et al. (2021). The displacement increments ($\Delta d$) associated with upward slopes of the force-displacement curves (not including the initial elastic part) are extracted, as shown in Fig. 2c, excluding those lower than the tolerance of the load cell (2.5 mN). The median value of these displacements per condition is considered the separation distance at damage initiation of the dissection, $\delta _{0} = median (\Delta d)$.

### Statistical analysis

Normal distribution of the data was checked using Shapiro-Wilk test (BenSaïda 2021). The significant difference among the response to dissection for each condition was studied by means of an independent one-tailed t-test. In cases of non-normal distribution, the Mann-Whitney test was used as comparison method. $p<0.05$ was established to indicate statistical significance. All data processing was performed with Matlab R2020a.

## Results

For both types of tests, the results of this study are displayed in plots that collect the force/width vs interface separation curves of all the samples per condition, as well as the average force/width throughout this separation. Moreover, mean and standard deviations of the dissection force/width and dissection energy are shown in respective tables and bar charts.

In all specimens, the experiment starts with an elastic deformation of the tongues, with no separation yet of the layers, as can be seen in Fig. 2.c. This phenomenon translates into a nonlinear upward slope at the beginning of the force/width vs displacement curves. Once the specimens start to dissect, the force reaches a plateau phase, which accounts for the dissection force of that specimen. This is the relevant data of this study and the part of the curves that will be displayed. This plateau force is not constant, but shows an irregular stability that is produced by the phenomenon of stick-slip tearing (Pasta et al. 2012).

### T-peel test

**Fig. 3:**
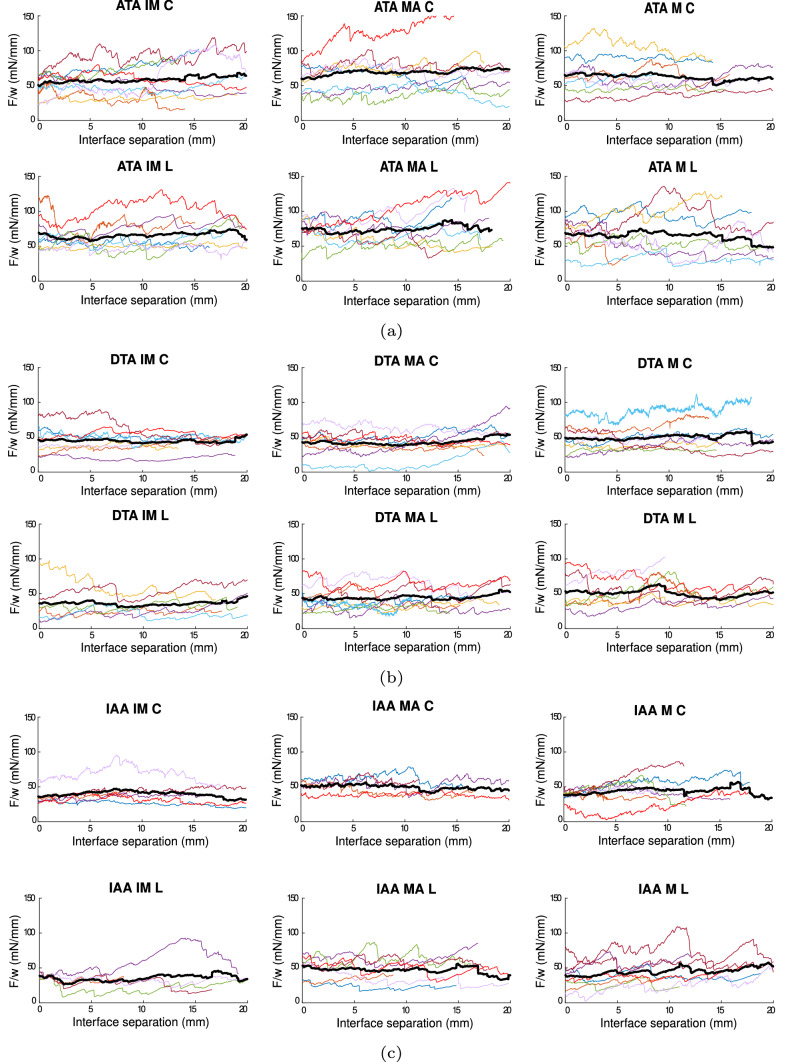
F/w (mN/mm) vs interface separation (mm) of the T-peel test performed in the specimens of the ATA (**a**), DTA (**b**) and IAA (**c**). For each region, the C direction is shown on top and the L one at the bottom. Separation of the IM is shown on the left, of the MA in the centre and the M on the right. Each individual is represented by one color. Data are *n* = 8, except for ATA IM C (*n* = 9), DTA IM L (*n* = 6), MA L (*n* = 9) and M L (*n* = 7) and IAA IM C and L (*n* = 6 and *n* = 5), MA C and L (*n* = 5 and *n* = 7) and M C (*n* = 7)

Figure 3 shows the force/width vs interface separation curves of all the specimens in the T-peel test, for the ATA, DTA and IAA. The results are displayed per orientation of the sample—longitudinal (L) or circumferential (C)—and per dissected interface—IM, MA or M. In the ATA, Fig. 3a, higher values of force/width in the dissection of samples in the longitudinal direction of the vessels can be observed for the separation of the two interfaces, the IM and MA, whereas the separation within the media shows similar values in both directions. The dispersion is high and appears similar for all cases. The force/width in the separation of the IM is generally lower than for the other two separations. In the DTA, Fig. 3b, there does not seem to be much difference in the values of force/width between the L and C directions in all three interfaces. The dispersion is again similar in every separation and direction, and so is the force/width needed to separate all three interfaces. Comparing with the ATA, the values of force/width of all cases, as well as the dispersion of results, are smaller in the DTA. In the IAA, Fig. 3c, force/width values in both directions are similar. The dispersion of results is higher in the L direction for the MA and M separations and, among interfaces, the dispersion of results is higher in the media. Separation of the MA required the highest force/width. Values of force/width are similar to those obtained in the DTA, but the dispersion of results is slightly lower.

**Fig. 4:**
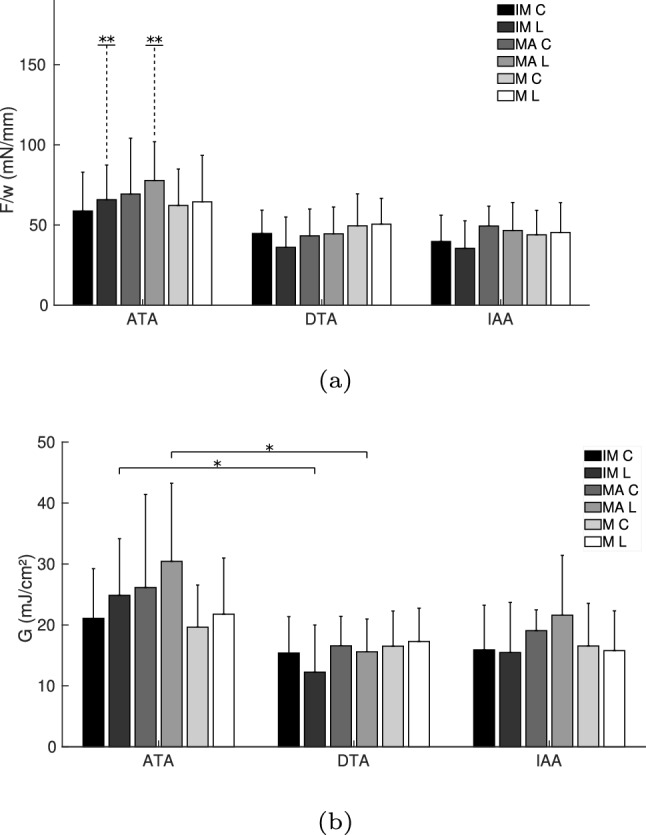
Averaged mean force/width and standard deviation (**a**) and dissection energy and standard deviation (**b**) of the T-peel test. *Statistically significant differences with a $p<0.05$. **Statistically significant difference, $p<0.05$, in the separation of the specified interfaces between the ATA and the DTA, as well as the ATA and the IAA, shown this way for graphical purposes

Table 2 shows the averaged mean force/width and standard deviation (mN/mm) of all conditions. The tendencies observed in Fig. 3 are supported with these data. Figure 4a displays this information graphically, indicating the cases with a significant difference. The dissection in the ATA presents the highest force/width values in all interfaces, with significant differences in the IM and MA in the longitudinal direction, when compared to the dissection in other parts of the aorta (IM L ATA–DTA & ATA–IAA, and MA L ATA–IAA with $p<0.05$; and MA L ATA–DTA with $p<0.01$). In the case of separating the MA, the lowest mean force/width was obtained in the DTA, whereas in the separation of the IM and M, the lowest mean force/width appeared in the IAA.

**Table 2:** Averaged mean force/width ± SD (mN/mm) of the T-peel tests. IM stands for intima-media, MA for media-adventitia and M for media. C and L are the circumferntial and longitudinal directions, respectively

	IM		MA		M	
$F_m$	C	L	C	L	C	L
ATA	58.72 ± 24.21	65.76 ± 21.67	69.27 ± 34.84	77.79 ± 24.15	62.18 ± 22.78	64.46 ± 29.01
	(*n* = 9)	(*n* = 8)	(*n* = 8)	(*n* = 8)	(*n* = 8)	(*n* = 8)
DTA	44.69 ± 14.54	36.03 ± 18.99	43.22 ± 16.72	44.53 ± 16.72	49.47 ± 19.91	50.53 ± 16.13
	(*n* = 8)	(*n* = 6)	(*n* = 8)	(*n* = 9)	(*n* = 8)	(*n* = 7)
IAA	39.73 ± 16.41	35.43 ± 17.14	49.41 ± 12.35	46.58 ± 17.44	43.85 ± 15.32	45.29 ± 18.61
	(*n* = 6)	(*n* = 5)	(*n* = 5)	(*n* = 7)	(*n* = 7)	(*n* = 8)

**Table 3:** Average dissection energy ± SD ($mJ/cm^2$) of the T-peel tests

	IM		MA		M	
$G_c$	C	L	C	L	C	L
ATA	21.08 ± 8.16	24.86 ± 9.29	26.15 ± 15.28	30.44 ± 12.81	19.64 ± 6.90	21.76 ± 9.23
DTA	15.41 ± 5.96	12.24 ± 7.76	16.57 ± 4.84	15.58 ± 5.40	16.52 ± 5.77	17.27 ± 5.48
IAA	15.92 ± 7.32	15.48 ± 8.21	19.05 ± 3.42	21.60 ± 9.81	16.53 ± 7.00	15.79 ± 6.52

**Table 4:** Separation distance at damage initiation (mm) of the T-peel tests

	IM		MA		M	
$\delta _0$	C	L	C	L	C	L
ATA	0.0809	0.0995	0.1633	0.1839	0.1499	0.0666
DTA	0.0702	0.2037	0.1500	0.1324	0.0501	0.1361
IAA	0.1147	0.1991	0.1174	0.2004	0.0998	0.1669

Table 3 shows the dissection energy per reference area obtained from the data of the T-peel tests and Fig. 4b displays this information graphically. Same as in the case of force/width values, the dissection energy in the specimens of the ATA is higher than in the other locations of the aorta, with significant differences in the IM and MA separation in the longitudinal direction between the ATA and DTA ($p<0.05$ and $p<0.01$, respectively). MA separation required less dissection energy in the DTA than in other locations, whereas there is no notable difference in the dissection energy of IM and M in the DTA and IAA. Table 4 shows the separation distance at damage initiation for the T-peel tests. It has been reported that this separation distance can be related to fracture toughness (Davis et al. 2016). However, no clear tendency can be found in these results.

### Mixed-mode peel test

**Fig. 5:**
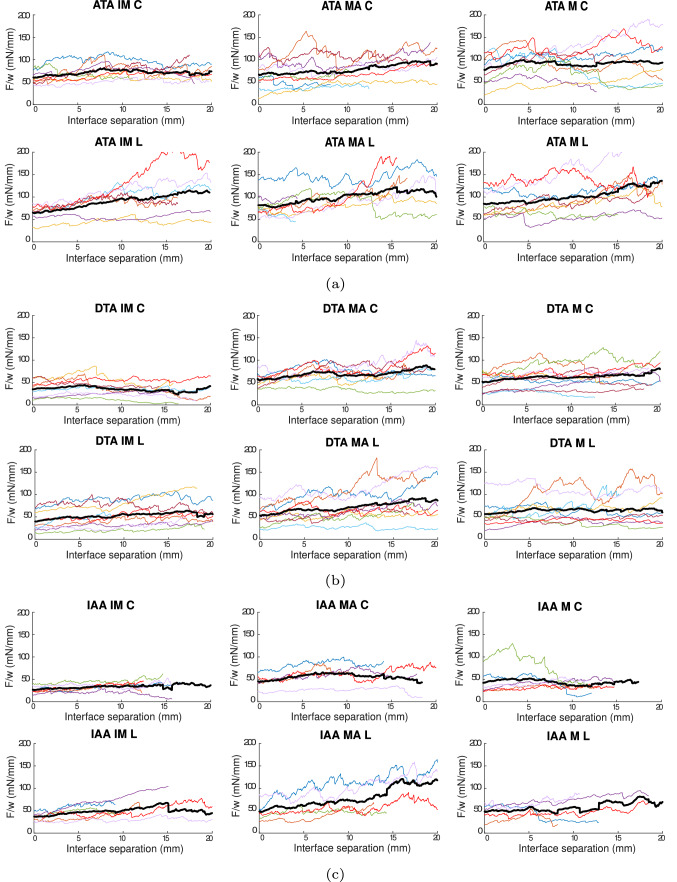
F/w (mN/mm) vs interface separation (mm) of the mixed-mode peel test performed in the specimens of the ATA (**a**), DTA (**b**) and IAA (**c**). For each region, the C direction is shown on top and the L one at the bottom. Separation of the IM is shown on the left, of the MA in the centre and the M on the right. Each individual is represented by one color. ATA IM C and L (*n* = 8 and *n* = 7), MA C and L (*n* = 9 and *n* = 8), M C and L (*n* = 9 and *n* = 8). DTA IM C and L (*n* = 8 and *n*=9), MA C and L (*n* = 8 and *n* = 9), M C and L (*n* = 10 and *n* = 9). IAA IM C and L (*n* = 6), MA C and L (*n* = 5), M C and L (*n* = 6 and *n* = 5)

Figure 5 shows the force/width vs interface separation curves of all the specimens in the mixed-mode peel test, for the ATA, DTA and IAA. In the ATA, Fig. 5a, the force/width to separate specimens in the longitudinal direction is higher in all interfaces than that in the circumferential direction. The dispersion of results is also somewhat higher in the longitudinal direction in general. As what happened in the T-peel test in this region, the force/width obtained in the separation of the IM interface is the lowest, as well as the dispersion of results in this interface. In the DTA, Fig. 5b, no differences can be appreciated among directions in terms of force/width, although the dispersion is higher in all three interfaces in the longitudinal direction. As what happens in the ATA, force/width values in the IM are also the lowest and the overall dispersion of results appears slightly lesser in this interface. Force/width values and dispersion of results are notably lower in the DTA than in the ATA, as what happened in the T-peel test. In the IAA, Fig. 5c, the force/width obtained in the longitudinal direction is higher than in the circumferential direction in the separation of the MA and in the M. The dispersion of results is similar among directions, except for the separation of the MA, where the L direction shows higher variability in the values. As what happens in the other regions of the aorta, the IM separation required the lowest force/width and shows the lowest dispersion. The values of force/width are similar to those obtained in the DTA, but the dispersion of results appears slightly lower. Compared to the results obtained in the T-peel test, the values of force/width obtained in the mixed-mode peel test are generally higher. This increased force/width is common (Gent and Kaang 1987; Zhang et al. 2012) and is thought to happen because of the bend of the sample in the mixed-mode peel test, while other explanations suggest that the combination of different modes of fracture (modes I and II) has some effect in this increased dissection force (Gent and Kaang 1987).

**Fig. 6:**
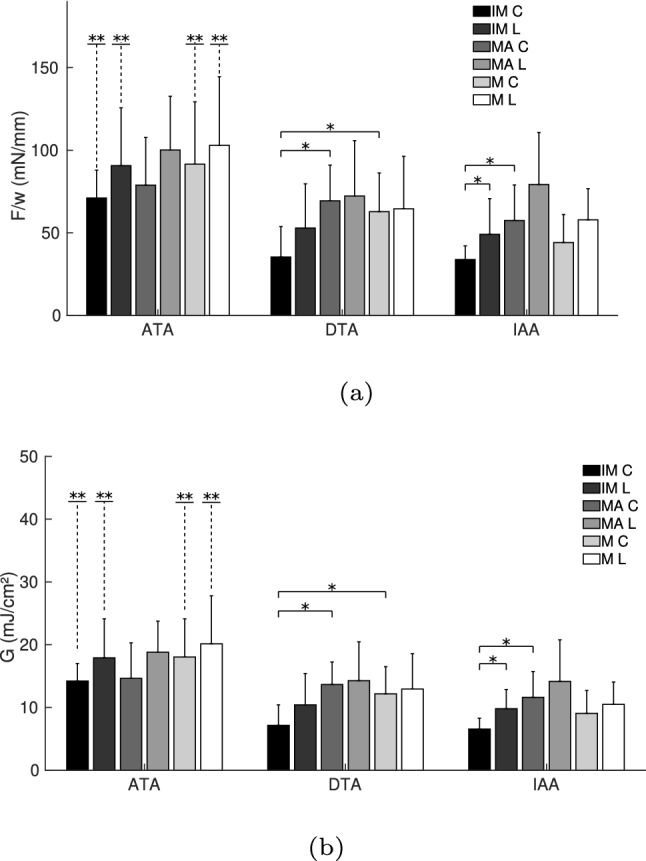
Averaged mean force/width and standard deviation (**a**) and dissection energy and standard deviation (**b**) of the mixed-mode peel test. *Statistically significant differences with a $p<0.05$. **Statistically significant difference, $p<0.05$, in the separation of the specified interfaces between the ATA and the DTA, as well as the ATA and the IAA, shown this way for graphical purposes

Table 5 shows the averaged mean force/width and standard deviation (mN/mm) of all conditions. Figure 6a displays this information graphically, indicating the cases with a significant difference. Similar to the T-peel test, the dissection of the ATA presented the highest forces among all layers, with significances in the cases of the IM and M dissections in both directions (IM C ATA–DTA and ATA–IAA with $p<0.001$; IM L ATA–DTA and ATA–IAA with $p<0.05$; M C ATA–DTA with $p<0.05$ and ATA–IAA with $p<0.001$; M L ATA–DTA and ATA–IAA with $p<0.05$). Higher force/width values to separate the longitudinal direction can be observed in the dissection of all layers and areas of the aorta, but there is statistical difference only in the case of the IM separation in the IAA ($p<0.05$). The IM separation shows the lowest values of force/width in all locations, with significances in the circumferential directions in the DTA (IM–MA with $p<0.01$ and IM–M with $p<0.05$) and IAA (IM–MA with $p<0.05$).

**Table 5:** Averaged mean force/width ± SD (mN/mm) of the mixed-mode peel tests

	IM		MA		M	
$F_m$	C	L	C	L	C	L
ATA	71.04 ± 16.92	90.69 ± 35.06	78.90 ± 28.80	100.16 ± 32.53	91.57 ± 37.77	103.03 ± 41.44
	(*n* = 8)	(*n* = 7)	(*n* = 9)	(*n* = 8)	(*n* = 9)	(*n* = 8)
DTA	35.34 ± 18.47	52.95 ± 26.75	69.37 ± 21.62	72.32 ± 33.49	62.80 ± 23.38	64.50 ± 31.88
	(*n* = 8)	(*n* = 9)	(*n* = 8)	(*n* = 9)	(*n* = 10)	(*n* = 9)
IAA	33.81 ± 8.25	49.10 ± 21.56	57.41 ± 21.58	79.29 ± 31.48	44.16 ± 16.95	57.90 ± 18.87
	(*n* = 6)	(*n* = 6)	(*n* = 5)	(*n* = 5)	(*n* = 6)	(*n* = 5)

**Table 6:** Average dissection energy ± SD ($mJ/cm^2$) of the mixed-mode peel tests

	IM		MA		M	
$G_c$	C	L	C	L	C	L
ATA	14.20 ± 2.80	17.90 ± 6.20	14.64 ± 5.65	18.81 ± 4.94	18.02 ± 6.10	20.14 ± 7.64
DTA	7.14 ± 3.27	10.40 ± 5.01	13.66 ± 3.57	14.27 ± 6.19	12.16 ± 4.33	12.93 ± 5.65
IAA	6.54 ± 1.75	9.79 ± 3.07	11.61 ± 4.11	14.16 ± 6.61	9.04 ± 3.66	10.50 ± 3.54

**Table 7:** Separation distance at damage initiation (mm) of the mixed-mode peel tests

	IM		MA		M	
$\delta _0$	C	L	C	L	C	L
ATA	0.1000	0.0572	0.0833	0.0550	0.0833	0.0495
DTA	0.1167	0.1001	0.0626	0.0831	0.1167	0.1167
IAA	0.0833	0.0999	0.0666	0.0341	0.0833	0.0526

Table 6 shows the dissection energy per reference area obtained from the data of the mixed-mode peel tests and Fig. 6b displays this information graphically. Following the tendency of the force/width and the results of the T-peel test, the dissection energy in the specimens of the ATA is the highest and in the IAA the lowest, with significances in the cases of the IM and M dissections in both directions (IM C ATA–DTA and ATA–IAA with $p<0.001$; IM L ATA–DTA and ATA–IAA with $p<0.05$; M C ATA–DTA with $p<0.05$ and ATA–IAA with $p<0.01$; M L ATA–DTA and ATA–IAA with $p<0.05$). Dissection energy for the specimens in the longitudinal direction was consistently higher than for the circumferential direction, with a significant difference in the case of IM separation in the IAA ($p<0.05$). Separation of the IM required less dissection energy than the other interfaces, and this difference was significant between the MA separation in the circumferential direction in the DTA ($p<0.01$) and IAA ($p<0.05$), and between the M separation in the DTA ($p<0.05$). Table 7 shows the separation distance at damage initiation for the mixed-mode peel tests.

### Histology

**Fig. 7:**
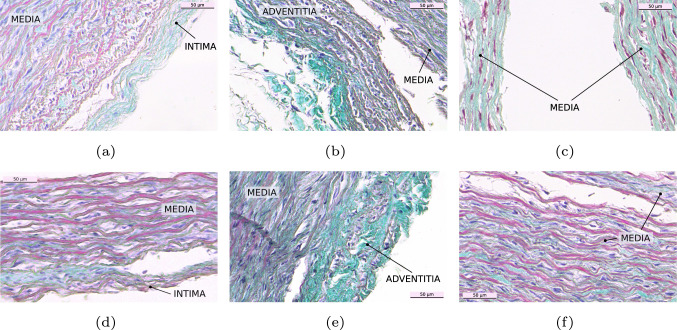
Masson’s trichrome histologies of the three dissected interfaces with the T-peel test in the C direction. **a**–**c** show separations in the DTA and **d**–**f** in the IAA. IM separation is on the left column, MA separation is on the middle and M separation on the right. The different layers are indicated on the images. Scale bar is 50 $\mu$m

Figure 7 shows different histologies performed on the delaminated interfaces of the DTA (a–c) and IAA (d–f) in the circumferential direction. The proper separation of the interfaces can be observed due to the distinct microstructural composition of the layers. Figure 7a and d show the IM interface. The higher amount of connective tissue (elastin) in the intima, in light green, helps differentiate this layer from the media, which is characterised by the presence of muscle fibres (red). The intima on the DTA is clearly visible, as well as the separation from the media with the internal elastic lamina. This distinction is not as clear in the IAA, but the intima can be differentiated by the higher amount of elastin (light green) on the outer area. Regarding the media and adventitia, Fig. 7b and e, both layers can be observed distinctively as the adventitial layer shows higher amounts of connective tissue (collagen, in light green). The gaps on the adventitia are fat tissue, also characteristic of this layer. Finally, Fig. 7c and f show the separation of the media. The circumferentially oriented muscle fibres can be perceived in the histologies.

## Discussion

Aortic dissections present different outcomes and severity depending on their location. Therefore, one key to understanding this disease comes from the study of the dissection properties of the artery and the variations throughout the vessel. To do so, entire porcine aortas, including the ATA, the DTA and the IAA were harvested and peel tests were performed to evaluate the dissection properties of the intima-media, media-adventitia and media within itself, in both vessel directions. Two dissection tests were performed to the specimens: the T-peel test, in which the separation happens in the perpendicular direction to the dissecting plane, and the mixed-mode peel test, in which the layers are separated in the direction parallel to the specimen. The T-peel test reproduces a mode I of fracture, a situation that does not accurately represent the dissection in the vessels (FitzGibbon and McGarry 2021; Haslach et al. 2018). However, most studies in literature that perform delamination tests in vessels have carried out this experiment and, therefore, it is an appropriate baseline for this study, as it will allow comparison of results. The mixed-mode peel test involves a mixed mode of fracture of modes I and II and is closer to the more complicated mechanisms that take place in the vessel. Both delamination tests are not the exact reproduction of the propagation of a tear in vivo. Nevertheless, interesting conclusions can be extracted from the results, as well as the dissection properties of the tissue, which can be calculated by generating computational models of these tests (Leng et al. 2018; Ríos-Ruiz et al. 2021; Wang et al. 2021)

The most notorious difference found in this study is the higher values of force/width and dissection energies that the specimens of the ATA present as opposed to the DTA and IAA for both tests. Tthese values of force/width and energies have also been found slightly higher for the DTA than for the IAA, mostly in the mixed-mode peel test, although with no statistical significance. Therefore, this suggests that the resistance to dissection tends to diminish when moving distally, which is contrary to the expectation as type A dissections—those that involve the ascending aorta—are more frequent (Evangelista et al. 2018; Hagan et al. 2000; Landenhed et al. 2015). These results agree with the studies in literature that have performed T-peel tests on the medial layer of healthy porcine aortas. Myneni et al. (2020) performed these delamination tests in specimens from the ATA and DTA. They obtained higher values of force/width for the specimens from the ATA (86–75.8 mN/mm) than for those from the DTA in both directions (62.7–48.4 mN/mm), and the values are similar to those obtained in this study. Similarly, Leng et al. (2018) performed T-peel tests on porcine IAAs. The values of force/width they reported are closer to the values in the DTA given by Myneni et al. (2020) (around 60 mN/mm). The values of energy given by both studies are not comparable since the methodologies used to obtained them were different. Wang et al. (2021) also performed T-peel tests on porcine DTAs and Noble et al. (2016) on thoracic aortas (not specifying between ascending or descending) and the values of force/width they obtained (72.27–46 mN/mm and 76.7–67.4 mN/mm) were lower than those of the ATA obtained by Myneni et al. (2020). Compared to the data in this study, the ranges of values match, but the force/width here obtained in the T-peel test is slightly lower. Regarding dissection energy, the values obtained by Noble et al. (2016) and Wang et al. (2021) agree with those obtained in this study of the DTA (18.3–15.2 $mJ/cm^2$ and 18.4–10.6 $mJ/cm^2$, respectively).

The propagation of dissection in different locations of the aorta had been studied with other methodologies. Roach and Song (1994) injected ink into the medial layer and controlled the pressure and volume needed to propagate tears in porcine aortas. They determined a uniform decrease of the tearing pressure and dissection energy while moving distally up to the upper abdominal aorta. However, when reaching the lower abdominal aorta, the energy increased dramatically. That decreasing tendency from the ATA to the upper abdominal aorta matches what was observed in this study.

The higher values of peeling force/width in the ATA have also been observed in healthy human aortas. Recently, Horný et al. (2022) and Sokolis and Papadodima (2022) studied the effect of location—among other parameters—in the dissection behaviour of the medial layer of the vessel via T-peel tests, obtaining these higher values of force/with in the ATA. Previously, Pasta et al. (2012) had performed T-peel tests on the medial layer of the human ascending thoracic aorta and obtained higher values of force/width in the delamination than Kozuń (2016) and Sommer et al. (2008), who performed the same tests on the human thoracic—presumably the descending region—and infrarenal abdominal aorta, respectively. Nevertheless, the specific values obtained in these studies do not completely match and cannot be directly compared to those of this work as they focused on human vessel. Apart from species, the differences between human and porcine tissue studies also arises due to age variations (Horný et al. 2022; Sokolis et al. 2017; Sokolis and Papadodima 2022). Human samples in most studies come from aged subjects, whereas the swines in the present study were comparably young, and dissection resistance has been shown to diminish with age (Horný et al. 2022; Sokolis and Papadodima 2022). In particular, the values of force/width of young human subjects reported by Horný et al. (2022) and Sokolis and Papadodima (2022) are in range with the results presented in this study.

The fact that dissections in the ATA are more frequent despite the higher dissection forces there needed to propagate a tear could be examined numerically. Through dissection models, the most crucial contributors of this disease could be clarified, whether they are the haemodynamic and mechanical forces in the ATA (Alimohammadi et al. 2015), the initiating factors that damage the wall (Beller et al. 2008) or the presence and evolution of aneurysms (Ho et al. 2017).

Regarding vessel anisotropy, in the aortic dissection studies that performed T-peel tests in the medial layer, force/width for specimens in the longitudinal direction of the vessel is generally higher and the dispersion is larger than for those in the circumferential direction (Horný et al. 2022; Leng et al. 2018; Myneni et al. 2020; Noble et al. 2016; Pasta et al. 2012; Sommer et al. 2008; Wang et al. 2021), although this tendency has not always shown a statistical significance. This happens because the dissection within the medial layer in the circumferential direction normally separates muscular fibres and lamellar structures, whereas the dissection in the longitudinal direction has to propagate throughout these structures. Sommer et al. (2008) observed the rougher surface of the longitudinal dissections compared to the circumferential ones with the help of histologies. In this study, this difference has not been found so predominantly, but is more prevalent in the results of the mixed-mode peel test, with even one statistically significant difference in the IAA, in the IM separation.

As for the separation distance at damage initiation, no clear differences are found throughout the experiments and the range of values agrees with literature (Wang et al. 2021). Wang et al. (2021) also did not find differences in this parameter on healthy tissue, but did when compared to purified elastin. In particular, $\delta _0$ was lower—although not statistically significant—in the dissection of purified elastin. This distance thus is less likely to vary unless substantial differences are imposed in the specimens.

Regarding the differences among interfaces, the main observation has been the easier propagation of a tear in the IM in terms of lower values of force/width and dissection energy in the DTA and IAA, more predominantly in the mixed-mode peel test. The results per interface in the ATA are fairly homogeneous. Tong et al. (2011) performed T-peel tests differentiating the dissection among layers in the human carotid bifurcation, while Kozuń (2016) and Kozuń et al. (2018) did the same on human thoracic aortas. In the three studies, the separation in the IM implied less dissection energy than the separation of the MA. This could be explained by less internal structures being dissected when these two different layers are separated, which are clearly set apart by the internal elastic lamina, making the dissection interface less rough and easier to split. Also, the smaller dissection energy and force/width to separate the intimal and medial layers accounts for the easier and favoured propagation of an initial dissection, while it slows down when it translates to the medial layer.

The possibility that the differences found in this study arise from testing different individuals and not different locations, interfaces or directions has also been evaluated, with no significant or prevalent tendencies found. It can only be pointed out that some aortas were more difficult to test than others, meaning that when tests failed in one aorta, they were more likely to keep failing in the same aorta. And also that, in some aortas, the differences between directions and interfaces were more marked and closer to the tendencies generally found in the literature. However, the number of full sets of samples is not enough to extract conclusions on this hypothesis.

The results here presented are not directly suitable to clinical practice and it is important to discuss some limitations. The main one is that this study has been performed in healthy porcine tissue. An already damaged endothelium or a degenerated media can be a trigger for aortic dissection (Fleischmann et al. 2022; Nienaber et al. 2016). Additionally, dissection is a common outcome of other pathologies like aneurysms (Angouras et al. 2019). Comparative studies between healthy and pathological tissue have shown the difference of dissection behaviour among them (Kozuń 2016; Kozuń et al. 2018; Pasta et al. 2012), as well as the the change in properties when the tissues are degraded (Noble et al. 2016; Wang et al. 2021), or even the differences with age (Horný et al. 2022; Sokolis and Papadodima 2022). Moreover, porcine aorta has been shown to provide different resistance to dissect than human aorta, in terms of peeling force/width and energy, therefore human models would require human tissue characterisation. Another limitation is the number of samples, as *n* = 9 may not be enough to completely avoid individual characteristics. Also, the aortas were not fresh and therefore their mechanical behaviour could have been affected by the freezing/unfreezing process.

However, some interesting conclusions can be obtained from this experimental study. Firstly, this study supports the assumption that the aorta has a location-dependent behaviour in terms of dissection properties. In particular, peeling force and energy decrease when moving distally through the aorta. These regional differences thus do not explain the major recurrence of aortic dissections in the ATA. Secondly, the different forces and energies obtained when separating the different layers are also shown. The dissection of the IM interface required the lowest force/width values in all locations of the vessel, accounting for the clear distinctive structure of these two layers. Therefore, this study suggests that dissection properties should be characterised in terms of location and interface of the vessel.

## Conclusions

Aortic delamination has been shown to require different levels of force/width throughout the different locations of the aorta, as well as in the separation of different interfaces. It is important to take account of this heterogeneity when modelling or studying this disease. Separation of the intima and media interface needed the less force/width and energy in the DTA and IAA, explaining the comparatively rapid propagation of the initial dissections throughout this interface until reaching the medial layer. Delamination in the ATA required the highest force/width in both experimental tests. Thus, the higher recurrence of aortic dissections in this area leads to the assumption that the mechanical and biological environment in the ATA is notably unfavourable for the development of this pathology.
